# An Incidental Diagnosis of Herlyn-Werner-Wunderlich Syndrome in a Young Female: A Case Report of a Rare Entity

**DOI:** 10.7759/cureus.53227

**Published:** 2024-01-30

**Authors:** Sravya Gudapati, Kamlesh Chaudhari, Apoorva Dave, Dhanajay Shinde

**Affiliations:** 1 Department of Obstetrics and Gynaecology, Jawaharlal Nehru Medical College, Datta Meghe Institute of Higher Education and Research (DMIHER), Wardha, IND; 2 Department of Radiodiagnosis, Datta Meghe Institute of Higher Education and Research (DMIHER), Wardha, IND

**Keywords:** herlyn–werner–wunderlich syndrome, renal agenesis, uterus didelphys, hematocolpos, ohivra syndrome

## Abstract

Obstructed hemivagina and ipsilateral renal anomaly (OHVIRA) syndrome, a rare genetic condition of the urogenital system, is characterized by distinctive features such as ipsilateral renal agenesis, obstructed hemivagina, and uterus didelphys. It is also referred to as Herlyn-Werner-Wunderlich (HWW) syndrome. Its clinical manifestations include dysmenorrhea, consistent abdominal pain, and infrequent periods. It is typically diagnosed after menarche. We report a case of a 20-year-old female who was admitted to the casualty ward following a road accident. She was Incidentally found to have uterine didelphys with hemorrhagic cystic lesion and left renal agenesis on ultrasonography (USG). She also had gallbladder stones, along with the findings mentioned above. Clinicians should exclude HWW syndrome in cases where uterine didelphys and unilateral renal agenesis coexist. Prompt identification and treatment of the condition can help avoid potential untoward pregnancy-related issues in the future.

## Introduction

Herlyn-Werner-Wunderlich (HWW) syndrome or obstructed hemivagina and ipsilateral renal anomaly (OHVIRA) syndrome is an unusual congenital condition characterized by the presence of a duplicated hemivagina, ipsilateral renal agenesis (one kidney failing to develop), and uterus didelphys (double uterus). Due to the occlusion of duplicated hemivagina, it usually manifests during adolescence and can cause symptoms such as pelvic pain, recurring infections, and abnormal menstrual bleeding [1,2]. It has also been associated with symptoms such as endometriosis, vaginal hemorrhage, pelvic inflammation, mucopurulent discharge, fever, abdominal pain, and vomiting [1]. Accidental diagnosis of HWW syndrome usually occurs when a patient seeks treatment for symptoms including recurring infections, pelvic pain, or atypical vaginal discharge. Clinicians may unintentionally find a double uterus, a duplicated hemivagina, and potentially ipsilateral renal agenesis during an examination or imaging tests like ultrasound or MRI, indicating the presence of HWW syndrome. Surgical interventions are preferred to address the anatomical defects and relieve symptoms in these cases. The goal is to remove the obstructive vaginal septum and, if feasible, preserve fertility while establishing appropriate drainage for the affected side.

## Case presentation

**Figure 1:**
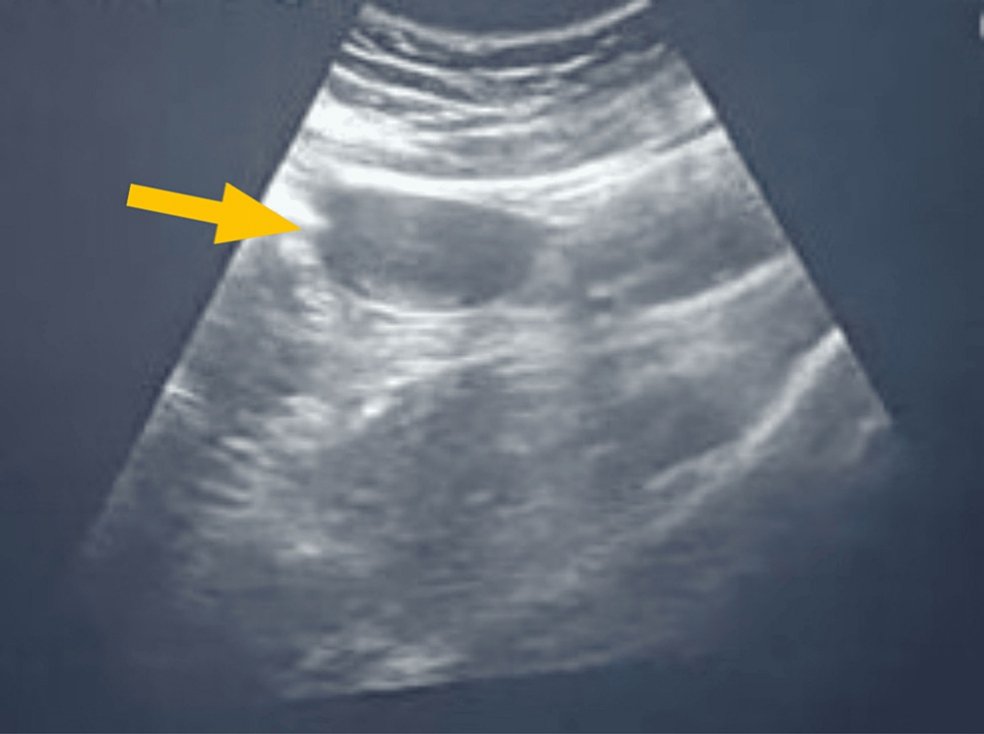
Sonography report of the patient depicting uterus with two horns

An unmarried 20-year-old female was admitted to the casualty department after a road accident. She gave a history of one episode of convulsion associated with postictal confusion. As part of the hospital protocol, traffic accident patients undergo Extended Focused Assessment with Sonography in Trauma (eFAST). Hence, a radiological screening by ultrasonography (USG) was performed, which revealed incidental findings such as a widely separated divergent uterine horn suggestive of the didelphic uterus, as depicted in Figure 1.

**Figure 2:**
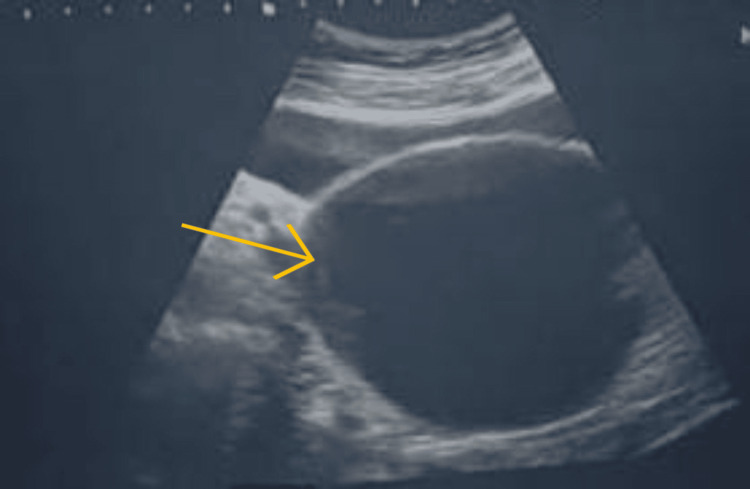
Patient's sonography report indicating hemorrhagic cystic lesion in between two uterine fundus

The presence of a didelphic uterus was accompanied by a hemorrhagic cystic lesion measuring 16 x 10 x 8 cm in between two uterine fundus, as depicted in Figure 2.

**Figure 3:**
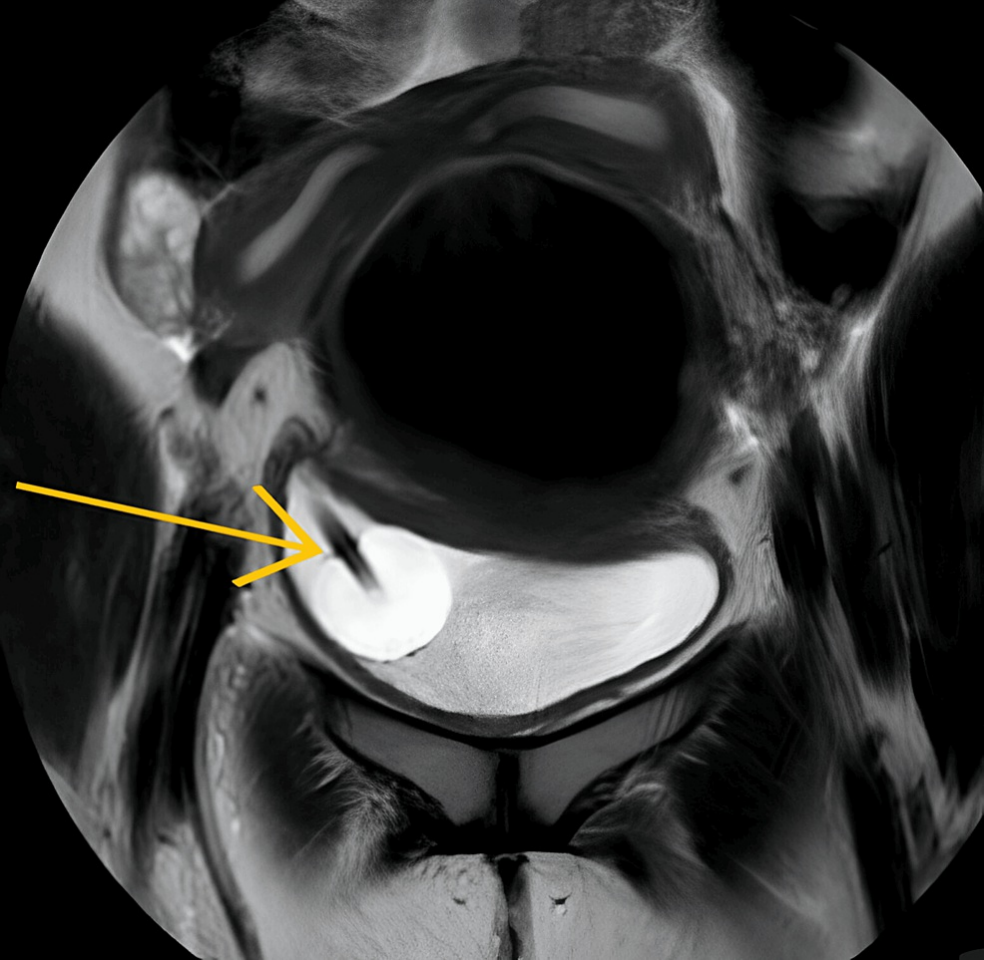
MRI showing uterus with two horns suggestive of bicornuate uterus MRI: magnetic resonance imaging

On USG, the right kidney was 14.2 x 6.2 cm in size, which led to the suspicion of compensatory hypertrophy or crossed fused ectopic kidney as the left kidney was not visualized. The gall bladder was over-distended with multiple calculi, the largest measuring 2 cm, indicating cholelithiasis. The patient further revealed that she had attained menarche at 14 years of age and has been having regular menstrual cycles of normal menstrual flow with cyclic pain in the left hypogastrium for the first two days of the cycle. An MRI was performed to rule out Mullerian abnormalities and to plan further management, and it revealed a didelphic uterus, as depicted in Figure 3.

**Figure 4:**
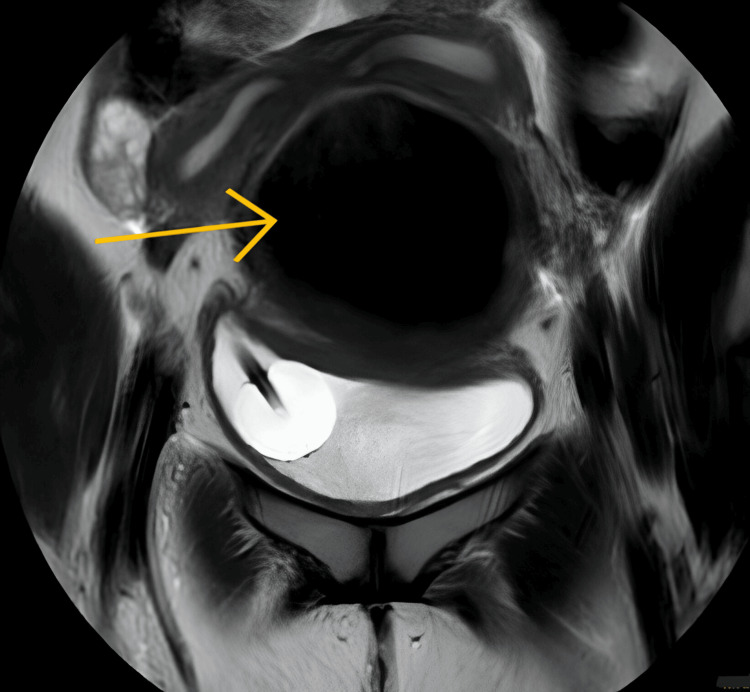
MRI showing hematocolpos and well-distended bladder with a Foley bulb in situ MRI: magnetic resonance imaging

MRI also showed a large altered signal intensity lesion of 89 x 103 x 138 mm in the pelvis with likely communication with the left uterine horn, suggestive of hematocolpos or hematometra, as depicted in Figure 4.

**Figure 5:**
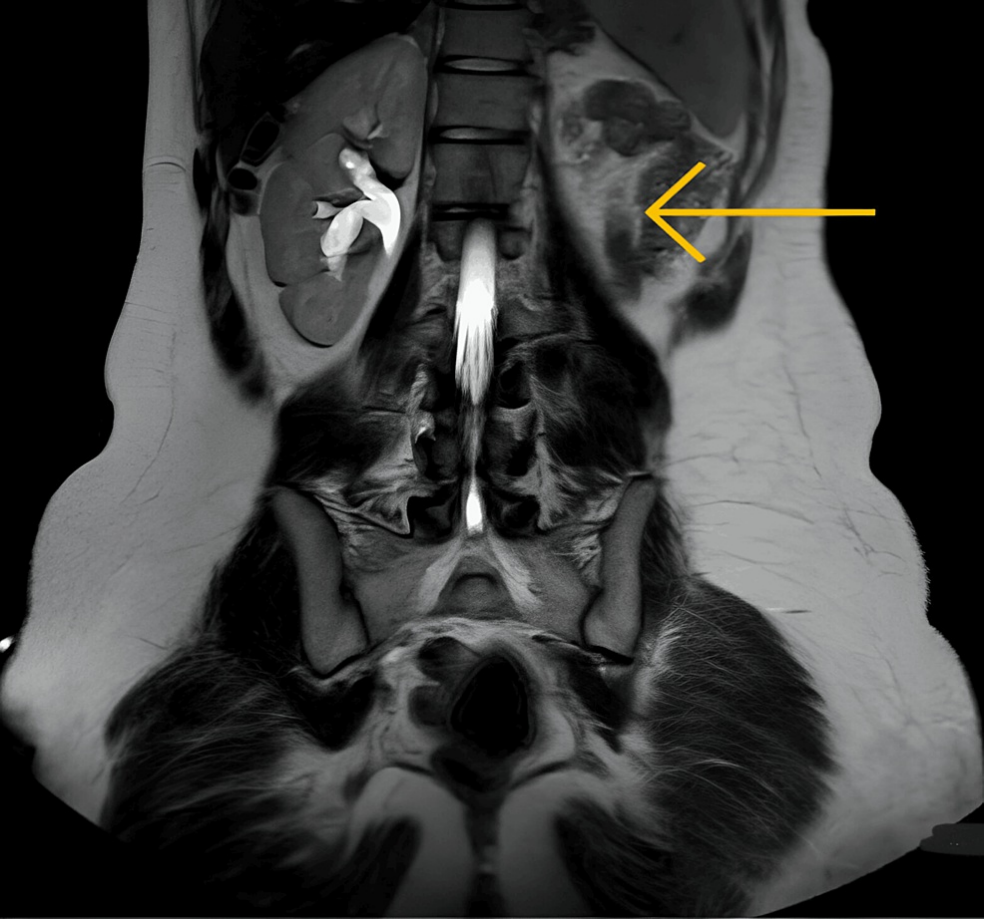
MRI showing empty left renal fossa MRI: magnetic resonance imaging

On MRI, the right kidney showed compensatory hypertrophy measuring 130 x 52 mm. The left kidney was not visualized in the left renal fossa or any ectopic site, suggestive of the absent left kidney, as depicted in Figure 5.

The patient's laboratory reports are presented in Table 1.

**Table 1:** Laboratory reports of the patient INR: international normalized ratio; LFT: liver function test; KFT: kidney function test; HCG: human chorionic gonadotropin

Parameter	Patient value	Normal range
Hemoglobin	12 gm%	13–17 gm%
Total leucocyte count	11,700/cu mm	4,000–10,000 cells/cu mm
Total platelet count	4.21 lakh/cu mm	1.5–4.1 lakh/ cu mm
Prothrombin time	10.6 seconds	<20 seconds
Activated partial thromboplastin time	28.5 seconds	29.5 seconds
INR	0.98	1-1.5
Random blood sugar	98 mg/dL	≤100 mg/dL
LFT	Within normal limits	
KFT	Within normal limits	
Thyroid-stimulating hormone	3.5 mIU/L	0.7–4.0 mIU/L
Urine analysis	Unremarkable	
Urinary beta HCG	Negative	

Based on the imaging results, vaginal septal resection under laparoscopic guidance was suggested to relieve hematocolpos. However, the patient opted for discharge against medical advice.

## Discussion

HWW syndrome was first defined by Herlyn and Werner in 1971 (renal agenesis with ipsilateral blind hemivagina). In 1976, Wunderlich recognized the presence of an isolated hematocervix associated with right renal agenesis and bicornuate uterus, and simple vagina [1]. "Mullerian duct abnormalities" refer to a broad spectrum of developmental malformations during fetal development due to defects in the septum's regression, fusion, or non-development. The mesonephric (also known as Wolffian) and paramesonephric (also known as Mullerian) ducts constitute the initial pairs of genital ducts in both male and female embryos. The paramesonephric ducts are created when the epithelium of the coelomic cavity invaginates longitudinally, as opposed to the mesonephric ducts, which originate straight from the mesonephros (primitive kidney).

The Mullerian ducts cross the ipsilateral Wolffian ducts ventrally in their caudal part before joining together in the middle to form the urogenital sinus. Two outflows, known as synovaginal bulbs, separate from the pelvic region of the sinus shortly after the paramesonephric ducts reach the urogenital sinus and give rise to the bottom two-thirds of the vagina. The uterine duct, which is paramesonephric in origin, gives rise to the upper portion of the vagina [2]. The most common developmental defect is renal agenesis, while cases of ectopic ureters, cystic renal dysplasia, duplication of the collecting system, and horseshoe- or pelvic kidney have also been described. Vercellini et al. have reported a higher prevalence of right-side abnormalities (66%) compared to left-side abnormalities, and they concluded that left-right asymmetry can develop before organogenesis, resulting in different morphogenesis on the right and left sides of the embryo [3]. A 10-year analysis of this anomaly revealed that dysmenorrhea (73%), a pelvic or paravaginal mass (71%), and an impacted right uterus and vagina (63.5%) were the most common symptoms in these patients [4].

However, our patient did not manifest any classic symptoms like dysmenorrhea, pelvic discomfort, a painful abdomen, and unpleasant mucopurulent vaginal discharge, as stated in various studies. The presence of a renal abnormality in a female should prompt clinicians to look for the genital tract. It had been missed previously in this patient and was diagnosed incidentally on ultrasound. A delayed diagnosis can lead to infertility, adhesions, endometriosis, and infectious issues triggered by chronic cryptomenorrhea [5]. The delays in diagnosis have been attributed to the lack of awareness and knowledge of this disorder among radiologists, gynecologists, urologists, nephrologists, pediatricians, and pediatric surgeons. In the setting of clinical suspicion of OHVIRA, imaging evidence can assist in establishing a diagnosis.

A robust medical understanding of Mullerian development abnormalities (MDAs) is possible when imaging methods identify unilateral renal agenesis at pre-pubescent or post-pubescent ages. These MDAs need to be examined with a transabdominal pelvic ultrasound with good visualization of the uterus and ovaries. The vaginal septum cannot be spotted on an abdominal or pelvic ultrasound. If the pelvic ultrasound imaging results in inaccurate visualization, the reproductive anatomy can be evaluated using a pelvis MRI with a magnetic field strength of 1.5 T or higher [6,7]. Regular menstruation can also contribute to a delayed diagnosis when it occurs in conjunction with a partial blockage of the vaginal outflow and a sluggish expansion of hematocolpos [8]. The initial task in managing OHVIRA syndrome involves performing a surgical procedure on the vaginal bulge and releasing the clogged hematocolpos. If the blockage comes from the vaginal septum, a vaginoplasty is often recommended. However, given that research has demonstrated the viability of pregnancy in a uterus that was previously blocked, hemi-hysterectomy of the obstructed uterus is no longer advised [9]. The uterus should be preserved regardless of the circumstances [10].

## Conclusions

HWW syndrome is an uncommon clinical presentation with scarce data available in the literature. Since prompt diagnosis requires a high index of clinical suspicion, doctors must be aware of this entity, even though it is not very prevalent. Since untreated cases of this abnormality might cause retrograde tubal reflux and endometriosis, an early and thorough diagnosis is essential. Timely diagnosis can help avoid adverse outcomes and aid in the management of associated symptoms. Lack of prompt treatment may also result in reduced fertility and obstetric problems later in life.
